# Mothers’ Breastfeeding Self-Efficacy after a High-Risk or Normal Pregnancy: A Greek Longitudinal Cohort Study

**DOI:** 10.3390/ejihpe14060119

**Published:** 2024-06-20

**Authors:** Panagiota Brani, Irina Mrvoljak-Theodoropoulou, Fani Pechlivani, Kleanthi Gourounti, Maria Iliadou, Ermioni Palaska, Panagiotis Antsaklis, Peter Drakakis, Maria Dagla

**Affiliations:** 1 Department of Midwifery, School of Health & Care Sciences, University of West Attica, 12243 Athens, Greece; pbrani@uniwa.gr (P.B.); fpechliv@uniwa.gr (F.P.); kgourounti@uniwa.gr (K.G.); miliad@uniwa.gr (M.I.); epalaska@uniwa.gr (E.P.); 2 Department of Psychology, National & Kapodistrian University of Greece, 15784 Athens, Greece; imrvoljak@hotmail.com; 3 First Department of Obstetrics and Gynecology, Medical School, National and Kapodistrian University of Athens, General Hospital “ALEXANDRA”,11528 Athens, Greece; pantsak@med.uoa.gr; 4 Third Department of Obstetrics and Gynecology, Medical School, National and Kapodistrian University of Athens, University Hospital “ATTIKON”,12461 Athens, Greece; pdrakakis@med.uoa.gr

**Keywords:** maternal self-efficacy, breastfeeding, educational interventions, socio-cultural factors, psychological support

## Abstract

Background: the objective of this longitudinal study (from pregnancy to the end of the sixth month postpartum) is to elucidate the association between maternal self-efficacy, defined as a mother’s confidence in her ability to breastfeed, and breastfeeding outcomes. Methods: This prospective cohort study was conducted among high-risk pregnant women (including those with conditions such as gestational diabetes, hypertension, pre-eclampsia, and other pathological medical conditions) and normal-risk pregnant women in Greece. The high-risk group included 164 women, while the normal-risk group comprised 154 women. Data were collected using validated psychometric scales, including the Breastfeeding Self-Efficacy Scale-Short Form, State-Trait Anxiety Inventory, Edinburgh Postnatal Depression Scale, Maternal Antenatal Attachment Scale, and Iowa Infant Feeding Attitude Scale. Results: Higher maternal self-efficacy was significantly associated with a longer duration and greater exclusivity of breastfeeding. A statistically significant relationship between the type of breastfeeding and the degree of breastfeeding self-efficacy was observed at multiple postpartum milestones: in the first and third 24 h postpartum, and at the end of the sixth week, third month, and sixth month postpartum. Conclusion: The findings underscore the critical role of maternal self-efficacy in breastfeeding success, influenced by individual psychological factors and broader socio-cultural contexts. Strengthening maternal self-efficacy is essential for improving breastfeeding outcomes.

## 1. Introduction

Self-efficacy, as defined by Bandura et al. [1] refers to the belief in one’s ability to successfully execute tasks and achieve desired outcomes. This construct is pivotal in behavioral performance, reflecting an individual’s perception of their capabilities rather than actual proficiency [2]. High self-efficacy fosters greater effort and positive emotional responses during task execution [1]. In the context of breastfeeding, self-efficacy critically influences a woman’s intention to breastfeed, the duration of breastfeeding, and the likelihood of exclusive breastfeeding [3]. A mother’s confidence in her breastfeeding abilities empowers her to overcome challenges, thereby enhancing both the quality and duration of breastfeeding. This underscores the necessity for interventions aimed at enhancing maternal self-efficacy [4,5].

A mother’s belief in her capacity to provide breast milk can significantly transform the breastfeeding experience into a positive one [6,7]. This confidence enhances maternal comfort and autonomy, influenced by an appreciation of the breastfeeding process [8], and strengthens the maternal-infant bond through feelings of unconditional affection [6]. Assessing self-efficacy impacts goal-setting, effort, and perseverance, influencing emotional resilience and responses to challenges [9]. Effective breastfeeding, considered a learned skill, necessitates a strong sense of self-assurance [10]. Postnatal support is crucial in boosting maternal self-confidence and facilitating breastfeeding [11]. Conversely, low self-efficacy can precipitate the early cessation of breastfeeding, with mothers perceiving breastfeeding as painful and challenging, leading to negative experiences and feelings of guilt [12,13,14,15,16,17,18].

Early breastfeeding difficulties, particularly within the first six weeks postpartum, often arise from perceived inadequate milk supply [19,20]. This perception undermines maternal confidence and can induce stress and anxiety, negatively impacting lactation through neurohormonal pathways [21,22]. Comprehensive education about breastfeeding processes and patterns can mitigate these issues, enhancing self-efficacy and reducing stress [23,24]. Maternal knowledge, attitudes, intentions, and social support are critical in shaping breastfeeding satisfaction and outcomes [25,26]. High self-efficacy is associated with greater satisfaction and more effective breastfeeding practices [26]. Socio-cultural backgrounds, family, and peer experiences significantly influence breastfeeding practices, either facilitating or hindering a mother’s ability to breastfeed [27,28].

Educational interventions and public health initiatives that promote breastfeeding can shift cultural perceptions and reduce associated stigma, thereby fostering prolonged breastfeeding [29,30,31,32]. Enhancing maternal self-efficacy through targeted support can improve breastfeeding practices and long-term health outcomes, underscoring the importance of comprehensive support systems [33,34].

The objective of this longitudinal study, spanning from pregnancy to the end of the sixth month postpartum, is to elucidate the association between maternal self-efficacy and breastfeeding outcomes, specifically in terms of exclusivity and duration. This investigation will encompass two distinct cohorts of pregnant women: those classified as high-risk pregnancies and those with low-risk pregnancies. Concurrently, this study aims to delineate whether maternal self-efficacy is modulated by psycho-emotional variables, including the following: (a) the extent of prenatal attachment between the expectant mother and the fetus, (b) the prenatal attitudes of the mother towards infant nutrition, (c) the degree of postnatal maternal-infant attachment, and (d) the presence of anxiety and depressive symptoms in the mother. This research seeks to contribute to the understanding of the interplay between psychological factors and breastfeeding practices, potentially informing interventions to support maternal and infant health.

## 2. Materials and Methods

### 2.1. Study Design

This research was conducted from the prenatal stage through to the end of six months postpartum at a public hospital in Attica, Greece. This study utilized a prospective cohort design.

### 2.2. Participant Recruitment and Eligibility Criteria

For this study, participants were recruited from two distinct groups, delineated by the medical risk associated with their pregnancies. This research was conducted using convenience sampling. The recruitment strategy was designed to encapsulate a broad spectrum of breastfeeding practices across varying maternal health conditions. The high-risk group included 164 pregnant women receiving specialized prenatal care within the hospital’s high-risk unit. This cohort was characterized by conditions such as gestational diabetes, hypertension, pre-eclampsia, fetal growth restriction, and significant risk of preterm labor, necessitating close medical supervision and intervention.

Conversely, the normal-risk group comprised 154 pregnant women undergoing routine prenatal monitoring at the hospital’s outpatient clinic, reflecting a standard pregnant population without the complications associated with higher-risk conditions.

Eligibility for participation required individuals to be at least 18 years old, fluent in Greek, and capable of providing informed consent. Participants needed to consent to participation and complete the study at the hospital, which involved regular follow-ups and adherence to the study’s evaluation processes. Exclusion criteria were implemented to maintain the study’s integrity and ethical standards. Specifically, non-Greek speakers were excluded to prevent language barriers that could impede full understanding and participation. Additionally, those unable to provide informed consent or follow-up, individuals with multiple pregnancies, and cases involving preterm delivery before 32 weeks or fetal/neonatal demise were excluded to ensure the study population accurately reflected the targeted demographic and risk profiles.

### 2.3. Data Collection Process

The data collection for this research extended over a period of 20 months, beginning in late May 2020 and concluding in January 2022. The process was carefully divided into five strategically defined phases, allowing for a detailed longitudinal analysis of breastfeeding intentions and practices from before birth to the endpoint of six months postpartum. This study commenced with a pilot phase, which was crucial for refining the research instruments and methodologies.

**Figure 1:**
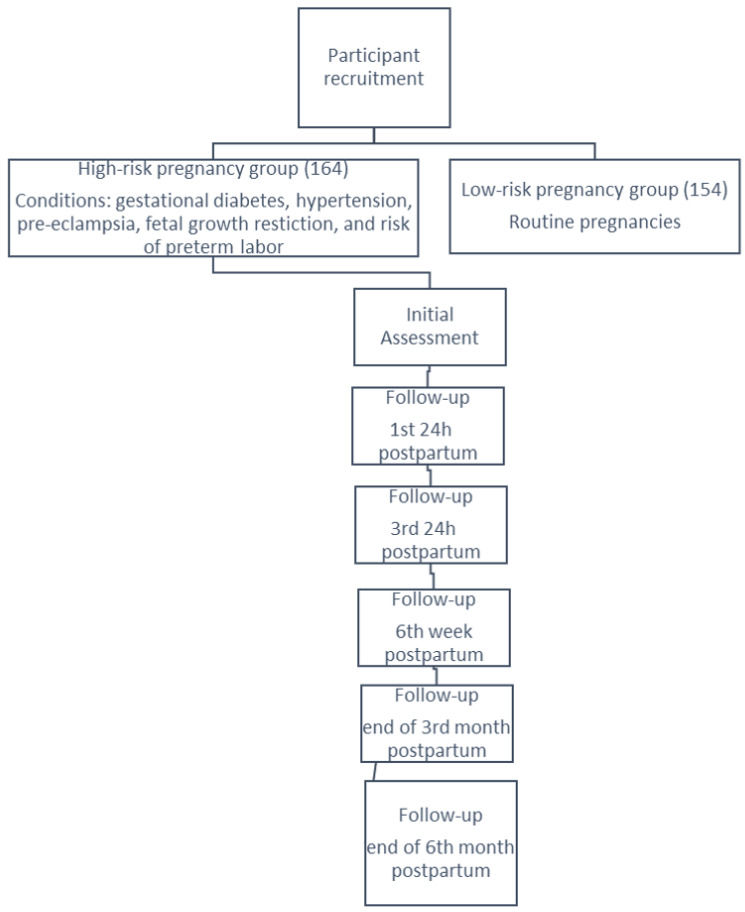
Flowchart of the data collection process.

This research was carried out in five distinct phases, each meticulously designed to collect critical data at various stages of the perinatal and postnatal periods (Figure 1).

Phase 1: In the first phase, both high-risk pregnant women during their prenatal hospitalization in the high-risk pregnancy unit and low-risk pregnant women attending regular outpatient clinics were asked to complete several questionnaires. These included assessments of socio-demographic and obstetric characteristics, and the State-Trait Anxiety Inventory (STAI), the Edinburgh Postnatal Depression Scale (EPDS), the Maternal Antenatal Attachment Scale (MAAS), and the Iowa Infant Feeding Attitude Scale (IIFAS) were used.

Phase 2: The second phase occurred on the 3rd to 4th day postpartum, during the participants’ stay in the postnatal wards. The administered questionnaires included follow-ups on post-delivery feeding methods, the short form of the Breastfeeding Self-Efficacy Scale (BSES-SF), State-Trait Anxiety Inventory (STAI) for state anxiety, EPDS for postnatal depression, and the Postpartum Bonding Questionnaire (PBQ). Women who had discontinued breastfeeding on the first day postpartum were exempted from completing the BSES-SF.

Phase 3: At the end of the puerperium, additional assessments were conducted via phone interviews or a specially designed online questionnaire form. These assessments included the outcome of breastfeeding practices and the same scales used in Phase 2.

Phase 4: At the end of three months postpartum, the fourth phase involved data collection through phone interviews or electronic forms. This phase focused on assessing ongoing breastfeeding status and the psychological and emotional well-being of the mothers using the same instruments as in the previous phases.

Phase 5: at the end of the six-month postpartum period, final assessments were conducted to evaluate the long-term outcomes of breastfeeding practices, maternal bonding, and psychological status using the same set of questionnaires.

Each phase was strategically designed to provide a comprehensive understanding of the breastfeeding journey, from prenatal intentions to postnatal practices, with a particular focus on the unique challenges faced by women with high-risk pregnancies.

Following the pilot, the data collection methodology adopted a hybrid approach to accommodate the diverse circumstances of the study participants and the challenges posed by the COVID-19 pandemic. This approach included both direct and indirect forms of engagement. Direct interactions were primarily conducted face-to-face during clinic visits, where participants could communicate directly with the researchers, providing real-time data and allowing for immediate clarification of any ambiguities. Indirect engagements were facilitated through telephone interviews and digital platforms, specifically using Google Forms for the submission of survey responses. This method was particularly useful for participants who were unable to attend in-person sessions due to health concerns, logistical issues, or the restrictions imposed by the pandemic. The combination of these methods not only maximized participation rates across a broad demographic, but also enhanced the depth and breadth of the data collected, contributing to a more comprehensive dataset that was robust and representative of the varied experiences of breastfeeding among the participants. Each phase of data collection was timed to align with critical milestones in the postpartum period, thereby capturing the dynamic nature of breastfeeding practices as they evolved over time. This structured approach ensured a systematic collection of data, which was essential for analyzing trends and changes in breastfeeding behavior from the prenatal period through to six months after childbirth.

### 2.4. Ethical Framework and Approvals

This research adhered to the ethical principles outlined in the Declaration of Helsinki, ensuring rigorous ethical standards in its execution. Before the study commenced, a comprehensive review was conducted by the Institutional Review Boards (IRBs), which thoroughly assessed the study’s design, participant engagement strategies, and overall methodology to ensure compliance with ethical norms pertaining to research involving human subjects. The detailed application for this study was designated protocol number 346 and was submitted on 20 May 2020. It received formal approval after rigorous scrutiny during the sixth session of the scientific council, which convened on 26 May 2020. This approval was crucial, as it confirmed the ethical integrity of this study and allowed for the initiation of data gathering processes under ethically approved guidelines.

### 2.5. Informed Consent Process

Prior to any data collection, detailed sessions were conducted with potential participants where they were informed about the aims, methodologies, potential risks, and benefits associated with this study. This process was integral to ensuring that participants were fully aware of the nature of this research and their role within it. Informed consent forms were meticulously crafted to underline the voluntary basis of participation, highlighting that participants could withdraw from the study at any point without any adverse effects. These forms also contained stringent assurances regarding the confidentiality of the data collected. Specific measures were outlined to anonymize participant data effectively and limit access to only those directly involved in the research, thereby safeguarding participant privacy. Additionally, the forms detailed the procedures in place for the secure handling and storage of data, reinforcing the commitment to maintain ethical standards and respect for participant rights throughout the research process. By ensuring that all ethical protocols were not only followed but clearly communicated to participants, this study upheld the highest standards of research ethics, emphasizing transparency, participant safety, and data integrity. These efforts ensured that this research provided meaningful insights while respecting the rights and welfare of all participants involved.

### 2.6. Classification of Breastfeeding Practices

In this study, breastfeeding practices were systematically categorized into two primary types: exclusive breastfeeding and mixed feeding. These classifications were based on established definitions to ensure consistency with global health guidelines and to facilitate accurate results. Exclusive breastfeeding was rigorously defined according to the World Health Organization’s (WHO’s) standards. Under this classification, an infant is considered to be exclusively breastfed when they receive only breast milk from their mother or a wet nurse, or expressed breast milk, and no other liquids or solids, not even water. The only exceptions allowed under this definition include oral rehydration solutions, drops, syrups of vitamins, minerals, medicines, and supplements prescribed by healthcare providers. This strict definition helps to maintain the integrity of this study’s data regarding infant feeding practices and ensures that the health outcomes associated with exclusive breastfeeding are accurately captured. Mixed feeding was defined as the administration of both breast milk and other nutritional sources to the infant. This category includes infants who are fed both breast milk and formula or other animal milks, as well as infants who receive breast milk along with solid or semi-solid foods before the recommended age of six months. This category is critical for assessing the dietary diversity that some infants experience and allows for an analysis of the potential impacts of combining breast milk with other dietary elements on health outcomes. By clearly defining these categories, this study aimed to delineate the boundaries between different feeding practices accurately. This classification is essential for evaluating the relationship between breastfeeding types and various health and developmental outcomes in infants. It also provides a reliable basis for recommendations on infant feeding practices to healthcare providers and policymakers based on scientifically gathered evidence [35,36].

### 2.7. Research Instruments and Psychometric Scales

In the pursuit of a comprehensive understanding of maternal psychological well-being, breastfeeding practices, and maternal-infant bonding, this study incorporated a carefully curated selection of validated psychometric instruments. These tools were chosen to evaluate specific psychological states and attitudes that are pertinent during the perinatal and postnatal phases, facilitating the collection of nuanced data essential for a multidimensional analysis of the participants’ psychological and behavioral profiles.

The psychometric scales used in this study have been validated for the target population, ensuring their reliability and accuracy. The State-Trait Anxiety Inventory (STAI) is well-established for measuring anxiety in both transient and enduring conditions [37]. This instrument differentiates between state anxiety, which captures the temporary feelings of anxiety influenced by the environment, and trait anxiety, which assesses the more stable aspect of anxiety proneness, such as general levels of stress and nervousness. In this study, the average Cronbach alpha coefficient was on the 1st Phase: 0.91, 2nd Phase: 0.93, 3rd Phase: 0.94, 4th Phase: 0.91, and 5th Phase: 0.92.

To detect symptoms of postnatal depression, the Edinburgh Postnatal Depression Scale (EPDS), introduced by Cox et al., was utilized [38]. This scale, consisting of ten items, asks respondents to rate their feelings over the past week, thereby facilitating the early identification of depressive symptoms during the postpartum period. The Edinburgh Postnatal Depression Scale (EPDS) is validated across various populations, with Greek studies setting cut-off points for postpartum depression at 8/9 (77% sensitivity and 68% specificity) and major depression at 12/13 (88% sensitivity and 86% specificity) [39]. In this study, the average Cronbach alpha coefficient was on the 1st Phase: 0.85, 2nd Phase: 0.88, 3rd Phase: 0.90, 4th Phase: 0.89, and 5th Phase: 0.89.

The emotional bond between a mother and her unborn child was assessed using the Maternal Antenatal Attachment Scale (MAAS), designed by Condon [40]. This scale measures the quality and intensity of the prenatal attachment, providing insights into the initial emotional connection of the mother with her expected child. The Maternal Antenatal Attachment Scale (MAAS) has demonstrated good internal consistency and validity in diverse studies, making it a reliable measure of prenatal attachment [41,42,43]. The average Cronbach alpha coefficient was 0.77 (1st Phase). Attitudes toward infant nutrition, particularly regarding breastfeeding versus formula feeding, were evaluated using the Iowa Infant Feeding Attitude Scale (IIFAS), developed by De la Mora [44]. It has proven to be a reliable tool for assessing attitudes toward infant feeding, with high internal consistency [45,46]. The average Cronbach alpha coefficient was 0.77 (1st Phase). The Breastfeeding Self-Efficacy Scale-Short Form (BSES-SF), created by Dennis, specifically measures a mother’s confidence in her ability to breastfeed [47]. This scale is crucial for identifying potential challenges to effective breastfeeding practices and determining the support needed to enhance lactation success. In this study, the average Cronbach alpha coefficient was on the 2nd Phase: 0.93, 3rd Phase: 0.95, 4th Phase: 0.94, and 5th Phase: 0.94.

Finally, the emotional bond between the mother and her newborn was examined through the Postpartum Bonding Questionnaire (PBQ), introduced by Brockington et al. [48]. This questionnaire helps in identifying any bonding disorders that may adversely affect the mother–infant relationship, crucial for targeting early interventions and support mechanisms. The Breastfeeding Self-Efficacy Scale-Short Form (BSES-SF) has shown high internal consistency globally, confirming its reliability in measuring maternal breastfeeding confidence [49,50,51]. The Postpartum Bonding Questionnaire (PBQ) has high internal consistency (2nd Phase: 0.89, 3rd Phase: 0.93, 4th Phase: 0.92, and 5th Phase: 0.93) and clinical relevance, making it a reliable measure of mother–infant emotional bonding [52,53,54].

These instruments were administered following standardized protocols to ensure the reliability and validity of the data collected. By employing these diverse tools, this study aimed to capture a holistic view of the maternal experience during the transition to motherhood, offering valuable insights into psychological health, attachment patterns, and feeding behaviors. This methodological approach enriches our understanding of maternal and neonatal well-being and informs interventions aimed at supporting mothers and infants during this critical period.

### 2.8. Statistical Analysis Strategy

The statistical analysis of the collected data was meticulously conducted using the Statistical Package for the Social Sciences (SPSS) software, version 22.0. Continuous variables were analyzed primarily through the application of Kruskal–Wallis and Pearson’s *r* analyses to assess the mean differences across groups. When assumptions for these tests were not met, appropriate non-parametric alternatives were considered to ensure the robustness of the results. In addition to basic descriptive and inferential statistics, a linear regression analysis was conducted to explore the relationships between continuous predictors and outcomes, providing a nuanced understanding of how various factors influenced the dependent variables on a continuous scale. Overall, the statistical strategy was designed to rigorously analyze and interpret complex relationships within the data, adhering to high standards of scientific inquiry to ensure that findings were both statistically significant and clinically relevant. This robust analytical approach provided the necessary framework to draw meaningful conclusions that could enhance maternal and neonatal health practices.

## 3. Results

Table 1 provides a detailed breakdown of the socio-demographic and perinatal characteristics distinguishing the high-risk pregnancy group from the low-risk pregnancy group. The data reveal an average age of 33.75 years (Standard Deviation [SD] = 5.48) for the high-risk pregnancy group and a slightly younger average of 31.69 years (SD = 6.00) for the low-risk pregnancy group. The level of education was high among both groups, with 44.5% (*n* = 73) of the high-risk pregnancy group and 48.1% (*n* = 74) of the low-risk pregnancy group holding university degrees. The majority of participants in both groups are married, comprising 83.5% (*n* = 137) of the high-risk pregnancy group and 90.9% (*n* = 140) of the low-risk pregnancy group, which reflects the supportive familial environments influencing perinatal care decisions. In terms of perinatal history, delivery methods varied significantly between the two groups. The high-risk pregnancy group had a higher rate of cesarean sections, at 79.9% (*n* = 131), compared to 48.1% (*n* = 74) in the low-risk pregnancy group, indicative of the medical interventions often necessary in high-risk pregnancies. Parity data show that the majority of participants were experiencing their first childbirth, with 54.3% (*n* = 89) in the high-risk pregnancy group and 56.5% (*n* = 87) in the low-risk pregnancy group. The timing of decisions regarding breastfeeding shows that a substantial number of women in both groups decided on their breastfeeding plans before becoming pregnant, with 76.2% (*n* = 125) in the high-risk pregnancy group and 81.8% (*n* = 126) in the low-risk pregnancy group pre-planning their feeding strategies. This indicates considerable forethought and commitment to infant nutrition. Moreover, the gestational age at birth showed significant differences, with 27.4% (*n* = 45) of the high-risk pregnancy group giving birth preterm (at or before 37 weeks), compared to only 2.6% (*n* = 4) in the low-risk pregnancy group, highlighting the high-risk pregnancy group’s complex risk factors and their potential impact on postnatal care and breastfeeding practices.

In the following analyses (Table 2), using Kruskal–Wallis statistical criterion, a relationship between the type of breastfeeding and the degree of breastfeeding self-efficacy (in the first and third 24 h postpartum, at the sixth week postpartum, and at the end of the third and sixth month postpartum) was estimated. A statistically significant relationship between the type of breastfeeding and the degree of breastfeeding self-efficacy was present in both groups of pregnant women (in the high-risk pregnancy group, in the first 24 h postpartum–BSES-*χ*^2^ = 33.392 and *p* = 0.004, in the third 24 h postpartum—BSES-*χ*^2^ = 53.904 and *p* < 0.001, at the sixth week postpartum—BSES-χ^2^ = 67.618 and *p* < 0.001, at the end of the third month postpartum—BSES-*χ*^2^ = 55.967 and *p* < 0.001, and at the end of the sixth month postpartum—BSES-*χ*^2^ = 48.119 and *p* < 0.001. In the low-risk pregnancy group in the first 24 h postpartum—BSES-*χ*^2^ = 15.441 and *p* < 0.001, in the third 24 h postpartum—BSES-*χ*^2^ = 10.989 and *p* < 0.001, at the sixth week postpartum—BSES-*χ*^2^ = 73.922 and *p* < 0.001, at the end of the third month postpartum—BSES-*χ*^2^ = 70.850 and *p* < 0.001, and at the end of the sixth month postpartum—BSES-*χ*^2^ = 26.201 and *p* < 0.001). It seems, in both groups, that mothers who breastfed exclusively during all the phases of the research, compared to those who breastfed non-exclusively, reported a higher degree of breastfeeding self-efficacy at each phase. Also, as it appears in both groups, through the analysis using the Spearman’s Rank correlation coefficient, a longer duration of breastfeeding is associated with a greater degree of breastfeeding self-efficacy in mothers from the hospital and up to the end of the sixth month postpartum. The highest correlation coefficients stand for breastfeeding self-efficacy at the sixth week postpartum and breastfeeding duration, while the lowest is between breastfeeding self-efficacy at the sixth month postpartum and breastfeeding duration (high-risk pregnancy group at the end of the sixth week postpartum—*ρ* = 0.605 ** and at the end of the sixth month postpartum—*ρ* = 0.313 **. In the low-risk pregnancy group at the sixth week postpartum—*ρ* = 0.565 ** and at the end of the sixth month postpartum—*ρ* = 0.212 *).

The next analyses concern the relationship between breastfeeding self-efficacy across all research phases. Related Pearson’s r correlation coefficients are presented in Table 3. It appeared, for both research groups, that breastfeeding self-efficacy correlates itself along all of the phases. As it appeared high in one phase, it appeared to be higher in another. The highest correlation coefficients are between breastfeeding self-efficacy at the sixth week postpartum and at the end of the third month postpartum (in the high-risk pregnancy group—*ρ* = 0.753 ** and in the low-risk pregnancy group—*ρ* = 0.884 **).

Table 4 presents the results of the linear regression analyses of both groups of pregnant women (high-risk pregnancy group and low-risk pregnancy group), regarding the relationship between breastfeeding self-efficacy at the sixth week postpartum and possible predictors related to a mother’s psycho-emotional state and levels of prenatal and postnatal bonding of a woman with the fetus and newborn, respectively. The percentage of variability explained by the models is statistically different from 0. In this linear regression analysis, the “dependent” variable was the degree of breastfeeding self-efficacy at the sixth week postpartum. As for the high-risk pregnancy group, the predictors appear to be the following: (a) the woman’s attitude during pregnancy towards feeding her child will follow (*β* = 0.225 and *p* = 0.008) and (b) the degree of the postnatal bond between mother and infant at the sixth week postpartum (*β* = −0.512 and *p* < 0.01). Particularly, a significant regression equation was found with *F* = 5.260, *p* < 0.001, *R* = 0.633, and *R*^2^ = 0.325. This model explains 32.5% of the variance of breastfeeding self-efficacy at the sixth week postpartum. Regarding the low-risk pregnancy group, the predictors are as follows: (a) the woman’s attitude during pregnancy towards feeding her child will follow (*β* = 0.189 and *p* = 0.002), (b) the state anxiety at the sixth week postpartum (*β* = −0.189 and *p* = 0.047), (c) the occurrence of postpartum depression symptoms in the third 24 h postpartum (*β* = −0.280 and *p* = 0.009) and at the sixth week postpartum (*β* = −0.257 and *p* = 0.016), and (d) the degree of the postpartum bond between mother and newborn in the third 24 h postpartum (*β* = −0.187 and *p* = 0.003) and at the sixth week postpartum (*β* = −0.373 and *p* < 0.001). And in this case, a significant regression equation was found with *F* = 16.591, *p* < 0.001, *R* = 0.798, and *R*^2^ = 0.598, and the model explains 59.8% of the variance of breastfeeding self-efficacy at the sixth week postpartum.

According to the results concerning the high-risk pregnancy group of the present research, it appears that the higher degree of breastfeeding self-efficacy at the sixth week postpartum is predicted by: (a) a more positive attitude of a woman in pregnancy towards the breastfeeding, and (b) a greater degree of postnatal mother–infant bonding at the sixth week postpartum. Regarding the group of pregnant women with physiological pregnancy, it was found that the higher degree of breastfeeding self-efficacy at the sixth week postpartum may be predicted by (a) a more positive attitude of the woman in pregnancy towards the breastfeeding, (b) a lower state anxiety of a mother at the sixth week postpartum, (c) lower scores on the EPDS scale that assesses the manifestation of postpartum depression symptoms, in the third 24 h postpartum and at the sixth week postpartum, and (d) greater degree of a mother’s postpartum bond with the newborn in the third 24 h postpartum and at the sixth week postpartum.

The results of the linear regression analyses, for both groups of pregnant women (high-risk pregnancy group and low-risk pregnancy group) included in this research, regarding the relationship between the degree of breastfeeding self-efficacy at the end of the third month postpartum and possible predictors related to a mother’s psycho-emotional state and levels of prenatal and postnatal bonding of the woman with the fetus and newborn, respectively, are presented in Table 4. The percentage of variability explained by the models is statistically different from 0. In the present linear regression analyses, the “dependent” variable was the degree of breastfeeding self-efficacy at the end of the third month postpartum. Regarding the high-risk pregnancy group, predictions seem to be (a) the woman’s attitude during pregnancy towards feeding her child will follow (*β* = 0.231 and *p* = 0.027) and (b) the manifestation of postpartum depression symptoms, at the end of the third month postpartum (*β* = −0.363 and *p* = 0.047). Concretely, a significant regression equation was found with *F* = 2.563, *p* = 0.003, *R* = 0.591, and *R*^2^ = 0.213, and the model explains 21.3% of the variance of breastfeeding self-efficacy at the end of the third month postpartum. As for the low-risk pregnancy group, the only predictor appears to be the woman’s attitude during pregnancy towards feeding her child will follow (*β* = 0.201 and *p* = 0.017). Also, a significant regression equation was found with *F* = 4.817, *p* < 0.001, *R* = 671, and *R*^2^ = 0.357. This model explains 35.7% of the variance of breastfeeding self-efficacy at the end of the third month postpartum.

The results concerning the group of women with high-risk pregnancies in the present research show that the higher degree of breastfeeding self-efficacy at the end of the third month postpartum is predicted by (a) a woman’s more positive attitude towards breastfeeding in the gestational period and (b) a lower score on the EPDS scale at the end of the third month postpartum. Regarding the group of pregnant women with physiological pregnancies, it was found that the higher degree of breastfeeding self-efficacy of a mother at the third month postpartum is predicted by a woman’s more positive attitude towards breastfeeding during pregnancy.

## 4. Discussion

This study provides a detailed examination of the profound impact of maternal self-efficacy on breastfeeding outcomes, highlighting the intricate interplay among psychological, educational, and socio-cultural factors. It emphasizes the critical importance of a mother’s confidence in her breastfeeding abilities, which is essential not only for initiating breastfeeding but also for sustaining it over extended periods. This confidence, stemming from a mother’s belief in her ability to successfully breastfeed, is fundamental to her breastfeeding journey. Self-efficacy influences various aspects of breastfeeding, from the initial decision to breastfeed to perseverance through challenges and responses to societal and cultural expectations. High self-efficacy is associated with greater resilience against common breastfeeding issues, such as perceived insufficient milk supply or latching difficulties, which are often cited as reasons for early cessation. By fostering strong self-efficacy, mothers are more likely to continue breastfeeding despite these hurdles, thereby enhancing both the duration and quality of breastfeeding [21,55,56].

Furthermore, the influence of self-efficacy extends beyond the individual to the broader socio-cultural context in which breastfeeding occurs. Cultures that support and encourage breastfeeding can enhance individual self-efficacy, creating a positive feedback loop that supports individual mothers and fosters a positive culture of breastfeeding within the community [57]. Educational interventions aimed at increasing maternal self-efficacy provide mothers with the knowledge and skills needed to manage breastfeeding challenges effectively. These interventions include prenatal classes simulating breastfeeding scenarios, postnatal support groups, and accessible resources for addressing common issues [58]. These educational efforts result in more informed and confident mothers better equipped to make decisions aligning with their breastfeeding goals [59,60].

This research substantiates the pivotal role of maternal self-efficacy in breastfeeding, extending and enriching the discourse established by Bandura [1] on the influence of self-belief on action execution towards desired outcomes. Self-efficacy, particularly within the context of breastfeeding, has emerged as a potent determinant of both the initiation and sustained practice of breastfeeding, consistent with prior research illustrating that high self-efficacy correlates with increased durations and exclusivity in breastfeeding [3].

Breastfeeding self-efficacy emerged as a pivotal factor in both our study and that of De Roza et al. [61]. Higher baseline scores on the Breastfeeding Self-Efficacy Scale-Short Form (BSES-SF) were strongly associated with improved breastfeeding outcomes. This suggests that interventions designed to enhance self-efficacy are critical for promoting EBF. Furthermore, De Roza et al. [61] found that a higher perception of insufficient milk significantly predicted EBF, which aligns with our findings that pre-planned breastfeeding strategies and elevated self-efficacy are essential for sustaining breastfeeding practices.

Our findings extend these observations by emphasizing the significance of self-efficacy throughout the breastfeeding journey, not merely at its initiation. High self-efficacy fosters a positive feedback cycle, where successful breastfeeding experiences enhance confidence, leading to more persistent and effective breastfeeding practices. This cyclical reinforcement is crucial for maintaining breastfeeding despite common physical and psychological challenges. Additionally, this study highlights how self-efficacy influences a mother’s response to breastfeeding challenges. Mothers with higher self-efficacy are more likely to view difficulties as manageable and seek out support and solutions, rather than feel overwhelmed. This proactive approach maintains a positive breastfeeding experience and reduces the likelihood of premature cessation. Furthermore, our results suggest that self-efficacy can mitigate the impact of societal and cultural pressures that often discourage prolonged breastfeeding. Mothers with strong self-efficacy are less likely to succumb to negative societal judgments and more likely to continue breastfeeding according to their goals and their babies’ health needs, rather than conforming to external expectations. This continuous impact transforms breastfeeding into a positive and enriching maternal experience, as observed by Polido et al. [6] and Rozett and Fragoso [7], who noted that maternal confidence could make breastfeeding profoundly positive, enhancing satisfaction and fortifying the maternal-infant bond. Takushi et al. [8] also linked the aesthetic appreciation of breastfeeding to increased maternal comfort and autonomy, highlighting self-efficacy’s role in facilitating these benefits.

The challenges associated with breastfeeding, particularly those perceived as painful or difficult [16], underscore the crucial role of self-efficacy. Our study confirms that low self-efficacy significantly predicts early cessation of breastfeeding, corroborating findings by Oliveira, Oriá and Ximenes [12], Haga et al. [13], and Abuchaim et al. [14], which indicate that mothers with low self-efficacy are three times more likely to discontinue breastfeeding prematurely. These insights underline the need for targeted interventions to bolster self-efficacy among mothers during both prenatal and postnatal phases, potentially mitigating challenges that lead to premature cessation.

The findings of our study align closely with those of Khresheh et al. [62], McQueen et al. [63], Pollard [64], and Prasitwattanaseree et al. [65], particularly in the domains of breastfeeding self-efficacy and exclusive breastfeeding (EBF) rates. Each study emphasizes the critical role of targeted educational and supportive interventions in improving breastfeeding outcomes among primiparous women. Our study noted significant differences in breastfeeding self-efficacy between mothers who exclusively breastfed and those who did not, which is consistent with the observations of McQueen et al. [63] and Prasitwattanaseree et al. [65]. Both studies demonstrated that enhanced self-efficacy through structured, continuous support significantly improves breastfeeding practices. McQueen et al. [63] highlighted that individualized support sessions, both in-hospital and via telephone, effectively sustained breastfeeding by reducing early discontinuation rates. Similarly, Prasitwattanaseree et al. [65] found that comprehensive antenatal and postnatal education sessions, combined with regular follow-ups, significantly boosted self-efficacy and EBF rates up to six months postpartum.

Our study, in conjunction with the findings of De Roza et al. [61] and Economou et al. [66], underscores the substantial impact of maternal education on breastfeeding outcomes. Higher educational attainment is consistently correlated with a greater likelihood of sustained exclusive breastfeeding (EBF). De Roza et al. [61] identified tertiary education as a significant predictor of EBF up to six months postpartum, while Economou et al. [66] observed that mothers with postgraduate education were more likely to maintain EBF compared to those with secondary education. These results are paralleled in our study, wherein mothers with higher education levels exhibited increased breastfeeding self-efficacy and prolonged EBF duration.

In terms of EBF rates, our study found that pre-planned breastfeeding strategies and higher self-efficacy were crucial predictors of sustained breastfeeding, echoing the findings of Pollard [64] and Khresheh et al. [62]. Pollard’s [64] research underscored the importance of continuous educational support, which led to significantly higher full breastfeeding rates at six months. Khresheh et al. [62] similarly emphasized that targeted educational interventions significantly improved breastfeeding knowledge, which is a critical component of maintaining EBF, although they did not find a significant difference in six-month EBF rates. Our study also identified higher rates of cesarean sections and preterm births in the high-risk pregnancy group, highlighting the additional challenges faced by high-risk pregnancies. This finding is in line with the broader literature, which stresses the necessity of tailored support for mothers undergoing complicated deliveries. While Khresheh et al. [62] and Pollard [64] did not specifically address high-risk pregnancies, their emphasis on the importance of targeted support aligns with the needs identified in our study. The mode of delivery and its impact on breastfeeding outcomes were highlighted in multiple studies. Both Alzaheb [67] and our study found that cesarean deliveries were associated with reduced EBF rates. This indicates that mothers who undergo cesarean sections may require additional support to initiate and sustain breastfeeding, suggesting the need for targeted interventions for this demographic.

Our study also expands the understanding of socio-cultural and educational factors influencing breastfeeding self-efficacy. Similar to Silva et al. [27], we found that the experiences and cultural values transmitted through family and friends critically shape a mother’s approach to breastfeeding. This is further supported by Awaliyah, et al. [26], who emphasize the profound impact of socio-cultural influences on a mother’s breastfeeding capabilities. The influence of work-related factors on breastfeeding was also significant. Alzaheb [67] and Dun-Dery and Laar [68] identified shorter maternity leave and maternal employment as substantial barriers to sustained EBF. Working mothers were less likely to practice EBF for six months, consistent with our study’s emphasis on the challenges these mothers face in balancing professional responsibilities with breastfeeding. This underscores the necessity for supportive workplace policies that extend maternity leave and create breastfeeding-friendly environments.

Education plays a crucial role in shaping breastfeeding outcomes through its direct impact on maternal self-efficacy. By providing mothers with accurate and comprehensive information about breastfeeding, educational interventions ensure that mothers are well-prepared for breastfeeding realities, from understanding physiological aspects to managing potential challenges [69,70]. This knowledge empowers mothers, fostering a sense of competence and control, which are key components of self-efficacy. Educational programs normalize breastfeeding challenges, reducing stigma and promoting open discussions about common issues such as latching difficulties, milk supply concerns, and breastfeeding pain. This openness helps mothers anticipate potential problems and equips them with strategies to address these effectively, thereby reducing anxiety and enhancing confidence. Education also reshapes societal norms and attitudes. Integrating breastfeeding education into broader health and prenatal classes can shift communities towards more supportive attitudes, making breastfeeding a shared responsibility rather than solely the mother’s burden. Such community-level changes are vital for creating an environment where mothers feel supported and valued, further boosting their self-efficacy.

The role of healthcare providers in educational interventions is crucial. Providers well-trained in breastfeeding education offer informational support, emotional encouragement, and practical tips during prenatal visits and postpartum follow-ups. Their ongoing support helps mothers adjust breastfeeding techniques, manage expectations, and build resilience against challenges [71,72].

Our study also highlights the substantial impact of prenatal and postnatal psychological factors on breastfeeding self-efficacy. The strong association between maternal attitudes towards breastfeeding and their self-efficacy levels emphasizes the necessity for supportive counseling and targeted interventions to positively influence maternal attitudes and address concerns related to breastfeeding. We should mention that one of limitations of this study is that the sample was not calculated by using a probability procedure. The issue was considered by interpreting the results in terms of possibility, not causality.

In summary, understanding the impact of maternal self-efficacy on breastfeeding outcomes provides valuable insights into supporting mothers in their breastfeeding endeavors. This understanding can guide healthcare professionals, policymakers, and community leaders in crafting interventions and policies that cultivate environments supportive of sustained breastfeeding practices.

## 5. Conclusions

This study elucidates the pivotal influence of maternal self-efficacy on breastfeeding outcomes and maternal satisfaction, providing a nuanced understanding of the underlying mechanisms that support successful breastfeeding practices. The results demonstrate an association between elevated self-efficacy levels and prolonged, exclusive breastfeeding durations. These findings contribute significantly to the body of knowledge on maternal and infant health, underscoring the necessity for targeted interventions aimed at enhancing maternal self-efficacy. The role of healthcare providers is critical in the delivery of these interventions. By addressing psychological and socio-cultural barriers to breastfeeding, healthcare professionals can create supportive environments that empower mothers. This study underscores the importance of comprehensive educational programs and supportive community and healthcare frameworks in fostering maternal confidence and capability in breastfeeding. Future research should continue to explore the dynamics of maternal self-efficacy and breastfeeding across varied cultural and healthcare contexts. Such research is essential for tailoring interventions that are both effective and culturally appropriate, thereby supporting breastfeeding mothers globally. The contributions of this study lay a foundation for the development of evidence-based policies and practices that enhance breastfeeding outcomes through the empowerment of mothers, ultimately improving health outcomes for both mothers and infants.

## Figures and Tables

**Table 1:** Demographic, perinatal, and breastfeeding characteristics.

	High-Risk Pregnancy Group		Low-Risk Pregnancy Group	
Demographic Characteristics	*N/M*	*%/SD*	*N/M*	*%/SD*
Age	33.75	5.48	31.69	6.00
Education				
Primary School	17	10.4	6	3.9
High School	51	31.1	53	34.4
Bachelor’s Degree	73	44.5	74	48.1
Master’s Degree/PhD	23	14.0	21	13.6
Total	164	100.0	154	100.0
Marital Status				
Married	137	83.5	140	90.9
Single	16	9.8	9	5.8
Divorced/Separated	2	1.2	-	-
Partnership Agreement	9	5.5	5	3.2
Total	164	100.0	154	100.0
Number of Children				
1	89	54.3	87	56.5
2	52	31.7	53	34.4
≥3	23	14.0	12	7.8
Total	164	100.0	152	98.7
Perinatal Characteristics				
Type of Delivery				
Vaginal	33	20.1	80	51.9
Cesarian section	131	79.9	74	48.1
Total	164	100.0	154	100.0
Time frame for making the decision to breastfeed				
Before pregnancy	125	76.2	126	81.8
In pregnancy/Postpartum	39	23.8	28	18.2
Total	164	100.0	154	100.0
Week of Labor Onset				
≥37th	119	72.6	150	97.4
<37th	45	27.4	4	2.6
Total	164	100.0	150	97.4

Note, *N*—the sample size, *M*—mean, and *SD*—standard deviation.

**Table 2:** Relationship between breastfeeding and the degree of breastfeeding self-efficacy.

	Breastfeeding in the First 24 h Postpartum				
High-Risk Pregnancy Group		*Ν*	*Mean Rank*	*χ*^2^(2)	*p ^a^*
BSES in the hospital	Exclusive Breastfeeding	18	71.08	10.989	0.004
	Non-exclusive Breastfeeding	57	49.93		
	Formula	27	41.76		
Low-risk pregnancy group					
BSES in the hospital	Exclusive Breastfeeding	70	87.00	15.441	<0.001
	Non-exclusive Breastfeeding	58	61.56		
	Formula	17	54.38		
**High-risk pregnancy group**	**Breastfeeding in the third 24 h postpartum**				
BSES in the hospital	Exclusive Breastfeeding	41	70.76	33.392	<0.001
	Non-exclusive Breastfeeding	52	39.58		
	Formula	8	24.00		
Low-risk pregnancy group					
BSES in the hospital	Exclusive Breastfeeding	91	92.25	53.904	<0.001
	Non-exclusive Breastfeeding	50	43.13		
	Formula	4	8.50		
	**Breastfeeding at the sixth week postpartum**				
High-risk pregnancy group					
BSES at the sixth week postpartum	Exclusive Breastfeeding	71	79.39	67.618	<0.001
	Non-exclusive Breastfeeding	35	31.71		
	Formula	11	14.18		
Low-risk pregnancy group					
BSES at the sixth week postpartum	Exclusive Breastfeeding	92	89.18	73.922	<0.001
	Non-exclusive Breastfeeding	42	29.57		
	Formula	3	2.00		
	**Breastfeeding at the end of the third month postpartum**				
High-risk pregnancy group					
BSES at the end of the third month postpartum	Exclusive Breastfeeding	68	65.21	55.967	<0.001
	Non-exclusive Breastfeeding	29	21.03		
	Formula	3	2.00		
Low-risk pregnancy group					
BSES at the end of the third month postpartum	Exclusive Breastfeeding	83	76.50	70.850	<0.001
	Non-exclusive Breastfeeding	29	22.26		
	Formula	6	4.33		
	**Breastfeeding at the end of the sixth month postpartum**				
High-risk pregnancy group					
BSES at the end of the sixth month postpartum	Exclusive Breastfeeding	61	57.77	48.119	<0.001
	Non-exclusive Breastfeeding	24	18.71		
	Formula	4	8.00		
Low-risk pregnancy group					
BSES at the end of the sixth month postpartum	Exclusive Breastfeeding	81	57.60	26.201	<0.001
	Non-exclusive Breastfeeding	18	21.28		
	Formula	1	1.00		
	**Breastfeeding duration ^b^**				
	High-risk pregnancy group		Low-risk pregnancy group		
BSES in the third 24 h postpartum	0.377 **		0.409 **		
BSES at the sixth week postpartum	0.605 **		0.565 **		
BSES at the end of the third month postpartum	0.499 **		0.459 **		
BSES at the end of the sixth month postpartum	0.313 **		0.212 *		

Note 1. ^a^—Kruskal–Wallis statistical criterion and ^b^—Spearman’s Rank correlation coefficient. Note 2. **. Correlation is significant at the 0.01 level (two-tailed) *. Correlation is significant at the 0.05 level (two-tailed).

**Table 3:** Pearson’s r correlation coefficients of maternal breastfeeding self-efficacy.

High-Risk Pregnancy Group	*1*	*2*	*3*	*4*
1 BSES in the third 24 h postpartum	-	-	-	-
2 BSES at the sixth week postpartum	0.397 **	-	-	-
3 BSES at the end of the third month postpartum	0.311 **	0.753 **	-	-
4 BSES at the end of the sixth month postpartum	0.379 **	0.438 **	0.729 **	-
Low-risk pregnancy group				
1 BSES in the third 24 h postpartum	-	-	-	-
2 BSES at the sixth week postpartum	0.319 **	-	-	-
3 BSES at the end of the third month postpartum	0.292 **	0.884 **	-	-
4 BSES at the end of the sixth month postpartum	0.311 **	0.585 **	0.680 **	-

Note, **. Correlation is significant at the 0.01 level (two-tailed).

**Table 4:** Linear Regression analyses for the relationship between the mother’s degree of breastfeeding self-efficacy at the sixth week postpartum and her attitude towards feeding her child in pregnancy, her psycho-emotional state, and the level of prenatal and postnatal bonding with fetus and newborn/infant.

	The Degree of Maternal Self-Efficacy in Breastfeeding at the Sixth Week Postpartum				
High-Risk Pregnancy Group	*b*	*S.E.*	*β*	*t*	*p*
(constant *α*)	37.016	19.320		1.916	ns
MASS in pregnancy (≥32 week)	0.029	0.174	0.014	0.166	ns
IIFAS in pregnancy (≥32 week)	0.422	0.155	0.225	2.723	0.008
STAI state in pregnancy (≥32 week)	0.099	0.140	0.080	0.703	ns
STAI state in the third 24 h postpartum	−0.115	0.142	−0.093	−0.812	ns
STAI state at the sixth week postpartum	−0.313	0.189	−0.189	−1.656	ns
EPDS in pregnancy (≥32 week)	0.072	0.325	0.026	0.221	ns
EPDS in the third 24 h postpartum	0.131	0.334	0.050	0.391	ns
EPDS at the sixth week postpartum	0.142	0.386	0.049	0.367	ns
PBQ in the third 24 h postpartum	0.007	0.165	0.004	0.041	ns
PBQ at the sixth week postpartum	−1.036	0.230	−0.512	−4.506	<0.001
	*R* = 0.633, *R*^2^ = 0.325, *F* = 5.260, *df* = 13, and *p <* 0.001				
Low-risk pregnancy group					
(constant α)	44.692	11.887		3.760	<0.001
MASS in pregnancy (≥32 week)	0.168	0.115	0.086	1.462	ns
IIFAS in pregnancy (≥32 week)	0.283	0.088	0.189	3.220	0.002
STAI state in pregnancy (≥32 week)	−0.108	0.135	−0.082	−0.798	ns
STAI state in the third 24 h postpartum	−0.004	0.136	−0.003	−0.026	ns
STAI state at the sixth week postpartum	−0.252	0.126	−0.189	−2.003	0.047
EPDS in pregnancy (≥32 week)	−0.014	0.228	−0.005	−0.060	ns
EPDS in the third 24 h postpartum	−0.721	0.270	−0.280	−2.667	0.009
EPDS at the sixth week postpartum	−0.740	0.302	−0.257	−2.448	0.016
PBQ in the third 24 h postpartum	−0.395	0.129	−0.187	−3.065	0.003
PBQ at the sixth week postpartum	−0.656	0.155	−0.373	−4.230	<0.001
	*R* = 0.798, *R*^2^ = 0.598, *F* = 16.591, *df* = 13, and *p* < 0.001				

Note, *R*—Pearson’s multiple correlation coefficient, *R^2^*—estimation of the explanatory power of the model, *F* criterion, *df*—degrees of freedom, *p*—probability of statistical error for the regression coefficients, and *ns*—nonsignificant.

## Data Availability

Data are contained within the article.
